# NK Cell Terminal Differentiation: Correlated Stepwise Decrease of NKG2A and Acquisition of KIRs

**DOI:** 10.1371/journal.pone.0011966

**Published:** 2010-08-06

**Authors:** Vivien Béziat, Benjamin Descours, Christophe Parizot, Patrice Debré, Vincent Vieillard

**Affiliations:** 1 INSERM UMR-S 945, Hôpital Pitié-Salpêtrière, Paris, France; 2 Université Pierre et Marie Curie (Paris-6), Paris, France; 3 Laboratoire d'Immunologie Cellulaire et Tissulaire, Paris, France; Centre de Recherche Public de la Santé (CRP-Santé), Luxembourg

## Abstract

**Background:**

Terminal differentiation of NK cells is crucial in maintaining broad responsiveness to pathogens and discriminating normal cells from cells in distress. Although it is well established that KIRs, in conjunction with NKG2A, play a major role in the NK cell education that determines whether cells will end up competent or hyporesponsive, the events underlying the differentiation are still debated.

**Methodology/Principal Findings:**

A combination of complementary approaches to assess the kinetics of the appearance of each subset during development allowed us to obtain new insights into these terminal stages of differentiation, characterising their gene expression profiles at a pan-genomic level, their distinct surface receptor patterns and their prototypic effector functions. The present study supports the hypothesis that CD56^dim^ cells derive from the CD56^bright^ subset and suggests that NK cell responsiveness is determined by persistent inhibitory signals received during their education. We report here the inverse correlation of NKG2A expression with KIR expression and explore whether this correlation bestows functional competence on NK cells. We show that CD56^dim^NKG2A^−^KIR^+^ cells display the most differentiated phenotype associated to their unique ability to respond against HLA-E+ target cells. Importantly, after IL-12 + IL-18 stimulation, reacquisition of NKG2A strongly correlates with IFN-γ production in CD56^dim^NKG2A^−^ NK cells.

**Conclusions/Significance:**

Together, these findings call for the reclassification of mature human NK cells into distinct subsets and support a new model, in which the NK cell differentiation and functional fate are based on a stepwise decrease of NKG2A and acquisition of KIRs.

## Introduction

NK cells are one component of the innate immune system, able to recognise various targets without specific sensitisation. They constitute a first line of defence and can kill infected and tumour cells. Mature NK cells are heterogeneous and differ in their proliferative potential, homing characteristics, functional capacities and responses to a wide range of cytokines [1], [2]. The complexity of the NK cell compartment is attributable to the vast network of receptors that are expressed on NK cell surfaces, which allow them to recognise target cells. These receptors can be either inhibitory or activating, and it is the balance between these opposing signals that dictates the functional status of the cells [3]. Around 10% of the NK cells in peripheral blood are CD56^bright^; this immunoregulatory subset produces a wide range of cytokines and chemokines after monokine stimulation, but its ability to kill target cells spontaneously is poor. In contrast, the remaining 90% of the NK cells, the CD56^dim^ cytotoxic NK cell subset, produces relatively lower cytokine levels, but possesses an abundance of cytolytic granules and can spontaneously lyse susceptible target cells [4].

The precise relationship between these two major NK cell subsets, CD56^bright^ and CD56^dim^, has long been debated. Several recent reports have finally shown quite convincingly that CD56^bright^ NK cells are very likely precursors of the CD56^dim^ subset: the shorter telomeres of the latter, in both peripheral blood and lymph nodes, imply that they are older than the former [5], [6]. In addition, Romagnani *et al* have shown *in vivo* that efferent lymph nodes contain a substantial proportion of CD56^dim^ KIR^+^ NK cells [6]. Very recently, Yu *et al* reinforced the concept of a transition from CD56^bright^ to CD56^dim^ cells, proposing CD94 as a developmental intermediary between these NK cell subsets [7]. Along with others, we have demonstrated that NK cells are the first lymphocytes appearing in patients recovering from hematopoietic stem cell transplantation (HSCT) and that these cells are constituted mainly from CD56^bright^ NK cells [8], [9], [10], [11].

Other and we have shown that CD56^dim^ NK cells from patients who have recently had hematopoietic transplantations transiently overexpress NKG2A, compared with cells from healthy donors [8], [10], [11]. In addition, CD56^dim^ NK cells from cord blood highly over-expressed NKG2A [12]. Altogether, these data suggest that the modulation of NKG2A expression among CD56^dim^ cells may define a crucial step of their differentiation. NKG2A is an inhibitory NK receptor that has been reported to form disulfide-linked heterodimers with invariant CD94. The NKG2A ligand has been identified as HLA-E, a non-classical MHC class-Ib molecule that is widely distributed among various tissues, exhibits relatively low surface expression, and has limited polymorphism [13]. The cross-linking of NKG2A by target cells bearing HLA-E results in NK cells inhibition through the phosphorylation of the two immunoreceptor Tyr-based inhibitory motifs (ITIMs) present in the intracellular tail of NKG2A [14].

Although it is well established that KIRs, in conjunction with NKG2A, play a major role in the NK cell education that determines whether cells will end up competent or hyporesponsive, the mechanisms underlying this education are still debated [15]. One hypothesis proposes that the engagement of self-MHC-specific inhibitory receptors directly renders NK cells functional, “licensing” them, so to speak. The “disarming” model, on the other hand, suggests that the hyporesponsiveness of NK cells lacking self-MHC-specific inhibitory receptors is a consequence of the persistent stimulation of previously competent cells. More recently a new “rheostat” model argues that the responsiveness of each NK cell is tuned by the input of inhibitory receptors [16], [17], [18].

Here we used a combination of complementary approaches that assess the kinetics of the appearance of each subset during development to examine the terminal stages of differentiation in the light of these hypotheses and to develop a detailed model that integrates our new insights with recent findings by others. All the observations from this study support the previous hypothesis that CD56^dim^ cells derive from the CD56^bright^ NK cell subset. Furthermore, this study provides the first phenotypic and functional analysis of mature CD56^dim^ subsets that focuses on the progressive decrease in NKG2A expression and the sequential acquisition of KIRs. We thus put forward a new model of terminal differentiation that starts at the CD56^bright^ stage and leads through the following stages: CD56^dim^NKG2A^+^KIR^−^CD56^dim^NKG2A^+^KIR^+^ CD56^dim^NKG2A^−^KIR^+^. Our data support the concept that terminally differentiated CD56^dim^NKG2A^−^KIR^+^ NK cells are fully competents, able to kill HLA-E^+^ targets, or alternatively CD56^dim^NKG2A^−^KIR^−^ hyporesponsive NK cells, due to impaired KIR acquisition.

## Materials and Methods

### Samples collection and treatment

All samples used in this study derived from anonymous individuals at the EFS (Etablissement Français du Sang), the National Blood Service. Written Informed consent was obtained for each donor. This study was approved by the institutional review board at Pitié-Salpêtrière Hospital (Paris, France). PBMC were isolated using Ficoll gradient immediately following collection. Cells were thereafter used for different tests.

### Flow cytometry

Five-colour fluorescent activated cell-sorting analysis was performed on freshly harvested PBMCs, or whole blood cells for post-UCBT. Cells were stained with the appropriate antibody cocktail: anti-CD3 (UCHT1; Beckman Coulter), anti-CD56 (N901; Beckman Coulter), anti-CD16 (3G8; Beckman Coulter or VEP13; Miltenyi biotec), anti-CD94 (HP-3D9; Becton Dickinson), anti-CD159a/NKG2A (Z199; Beckman Coulter), anti-NKG2C (134591; R&D systems), anti-CD85j/ILT2 (HP-F1; Beckman Coulter), anti-CD27 (B1.49.9; Beckman Coulter), anti-CD62L (MEL-14; Becton Dickinson), anti-CD57 (S-HCL-1; Becton Dickinson), anti-Siglec-9 (191240; R&D Systems) and anti-FCRL6 (2H3; BioLegend). Global KIR analysis was performed with an anti-KIR antibody mix: anti-KIR2DL1/KIR2DS1 (EB6B; Beckman Coulter), anti-KIR2DL2/KIR2DL3/KIR2DS2 (GL183; Beckman Coulter) and anti-KIR3DL1/KIR3DS1 (Z27; Beckman Coulter). Single KIR analysis was performed by subtraction to discriminate between KIR2DS1 and KIR2DL1, in the presence of anti-KIR2DL1 (143211; R&D Systems) and anti-KIR2DL1/KIR2DS1. Similarly, KIR2DL2 and KIR2DL3 were discriminated with anti-KIR2DL3, (180701; R&D Systems) and anti-KIR2DL2/DL3 (DX27, Miltenyi Biotec). Other single KIRs were detected with anti-KIR3DL1 (DX9; R&D Systems), anti-KIR2DS4 (179315; R&D Systems) or anti-KIR2DL5 (UP-R1; E-Biosciences). FACS lysing solution (Becton Dickinson) was used to lyse erythrocytes for whole blood staining. Cells were permeabilised for intracellular staining with cytofix/cytoperm kit (Becton Dickinson) and then stained with anti-granzyme-K (GM6C3; Santa Cruz) or anti-IFN-γ (B27, BD Biosciences) mAbs. IFN-γ staining was performed after overnight incubation in the presence of 10 ng/ml IL-12 (R&D Systems) plus 100 ng/ml IL-18 (R&D Systems).

### Microarray analysis

10^4^ cells of CD56^bright^CD16^−^, CD56^dim^NKG2A^+^ and CD56^dim^NKG2A^−^ NK cell subsets from four different healthy donors were sorted by FACS. Purified cells were collected and then resuspended in SuperAmp™ Lysis Buffer (Miltenyi Biotec, Cologne, Germany). The Agilent 2100 Bioanalyzer System (Miltenyi Biotec) was used to control total RNA quality. Gene expression analysis was performed with the Human Whole Genome Oligo Microarray (44 K), as a commercial service by Miltenyi Biotec, to reveal a gene expression profile for each NK cell subset of every individual donor. The Agilent Feature Extraction Software was used to read out and process the microarray image. Data were then normalised by dividing each reporter signal with the median signal intensity of the whole microarray, and then filtered to remove noisily or arbitrarily regulated genes with expression levels close to background. Reporters with expression intensities of less than 10% of the array median were removed from the dataset. Thus 33,867 of the 41,000 genes with strong reliable signals were sorted and then analysed with TM4 software [19]. Complete linkage hierarchical clustering was then performed on the significantly modulated genes (p<0.01), with one-way ANOVA. Each cluster was then individually used for ontogenetic analysis using Panther-BP (in the gene ontology section) of DAVID software [20], [21]. All microarray data are MIAME compliant and raw data have been deposited in the MIAME compliant database ArrayExpress (accession number: E-MEXP-2798).

### Cell sorting

Whole NK cells from freshly isolated PBMCs were negatively purified with a MACS NK cell isolation Kit (Miltenyi biotec). The various subsets of NK cells were sorted with a BD FACS-Aria (Becton Dickinson) cell sorter from purified NK cells in the presence of anti-CD3 (UCHT1; Beckman Coulter), anti-CD56 (N901; Beckman Coulter), anti-NKG2A (Z199; Beckman Coulter), and anti-CD16 (VEP13, Miltenyi). Post-sorting purities were routinely >96%. NK subsets were then used for the microarrays and for IFN-γ production assays.

### Degranulation assays

Degranulation assays used CD107a detection, as previously described [11]. PBMC were resuspended at a 1∶1 ratio in the presence of target cells and anti-CD107a mAb (H4A3; Becton Dickinson). After 1 h incubation, monensin was added at 2 mM for an additional 4 h of incubation. Degranulation was assayed against MHC class-I-deficient K562, 721.221 target cells, and LCL-221-AEA, which expresses the HLA-E*0101 allele [22]. Antibody-dependant cell cytotoxicity assays were performed against CD20^+^ RAJI cells in the presence of 1 µg/ml of anti-CD20 (rituximab, Roche).

### Statistical analysis

Mann Whitney tests were performed for individual comparisons of two independent groups. Wilcoxon tests were performed for individual comparison of the paired groups. Repeated measures ANOVA with Tukey post-test for P-value calculation were performed for multiple comparisons assays. Statistical analysis used Prism 5 software (GraphPad Software, San Diego, CA). *P* value less than 0.05 were considered as significant. 1* = p<0.05; 2* = p<0.01; 3* = p<0.001.

## Results

### Phenotypic and transcriptomic profile of CD56^bright^, CD56^dim^NKG2A^+^ and CD56^dim^NKG2A^−^ subsets

**Figure 1:**
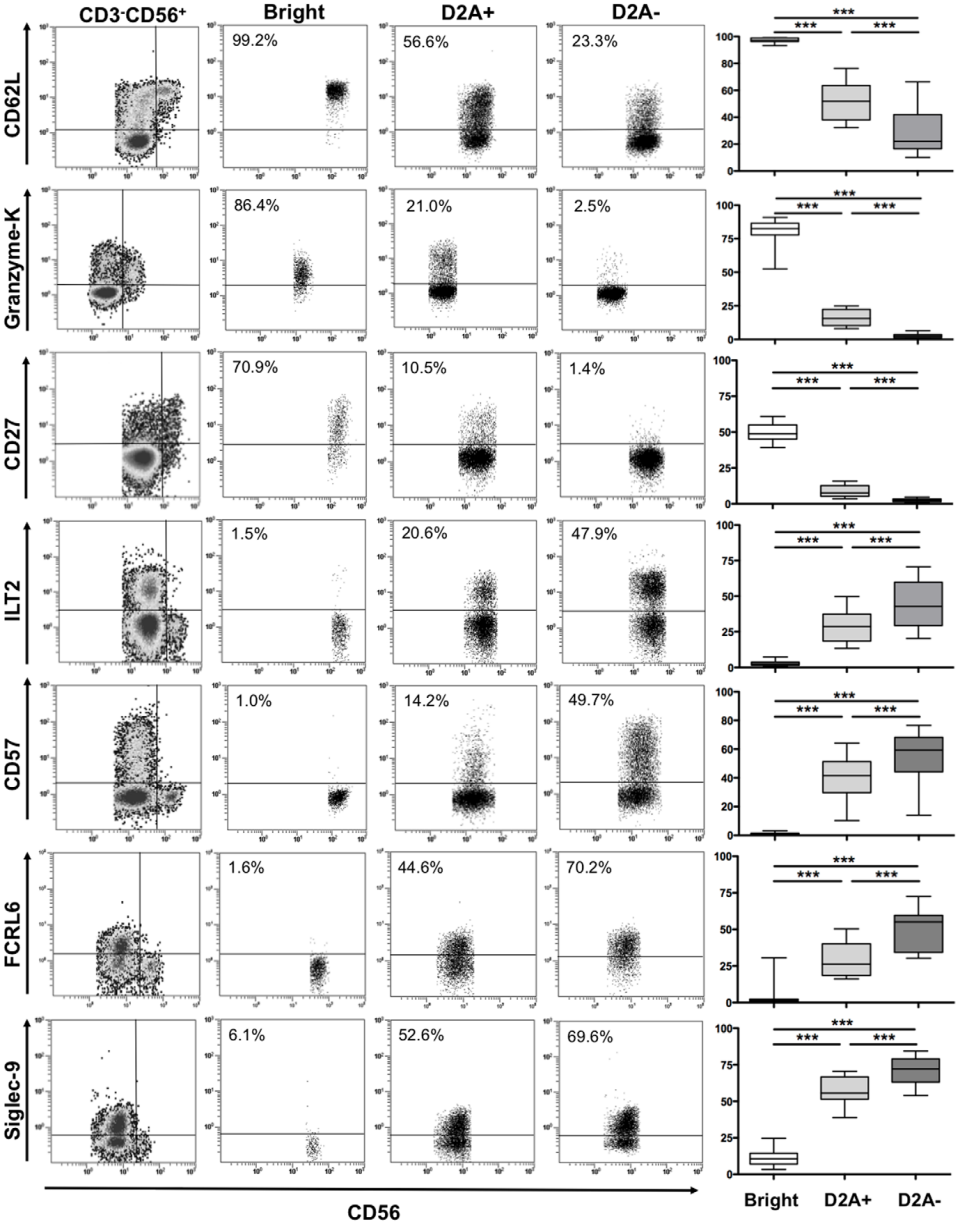
Phenotype patterns of CD56^bright^ (Bright), CD56^dim^NKG2A^+^(D2A+) and CD56^dim^NKG2A^−^(D2A−) NK cell subsets from healthy controls. Representative samples (left panels) and box and whiskers plots (right panels) are presented for each marker from at least 16 healthy controls. *: p<0.05; **: p<0.01; ***: p<0.001.

In order to better characterize the last steps of NK cell differentiation, we analysed the expression patterns of NK receptors and other maturation molecules on CD56^bright^, CD56^dim^NKG2A^+^, and CD56^dim^NKG2A^−^ cells from healthy controls by flow cytometry. As shown in Fig. 1, CD62L, granzyme-K and CD27 were highly expressed in CD56^bright^ NK cells, but more importantly their expression progressively decreased in CD56^dim^NKG2A^+^ and CD56^dim^NKG2A^−^ NK cells. Inversely, CD57, ILT2, FCRL6 and Siglec-9 progressively accumulated when looking sequentially these subsets. In contrast, other NK markers distinguished only CD56^bright^ and CD56^dim^. These included Pen5, CD8α, perforin and granzyme-B, which were expressed mainly on CD56^dim^ cells, independently of NKG2A expression. NKp44, CCR7, CD127 and CD117 were expressed almost exclusively on CD56^bright^ NK cells (data not shown). Finally, these three NK cell subsets were indistinguishable for the other receptors tested, including NKp30, NKp46, NKG2D, 2B4, LAIR-1, DNAM, CD11a and CD11b (data not shown). Of note, NKG2A is covalently associated with CD94 to form heterodimers and, as expected, expression of NKG2A and of CD94 was highly correlated (Figure S1). However, CD94 expression is also linked to that of NKG2C, an activating NK receptor that, like NKG2A, recognises HLA-E [23]. NKG2C is poorly expressed on NK cells from healthy donors. The CD56^dim^ NK subset expressed NKG2C and NKG2A mainly separately while the CD56^bright^NKG2C^+^ subset co-expressed NKG2A (Figure S1) [24].

**Figure 2:**
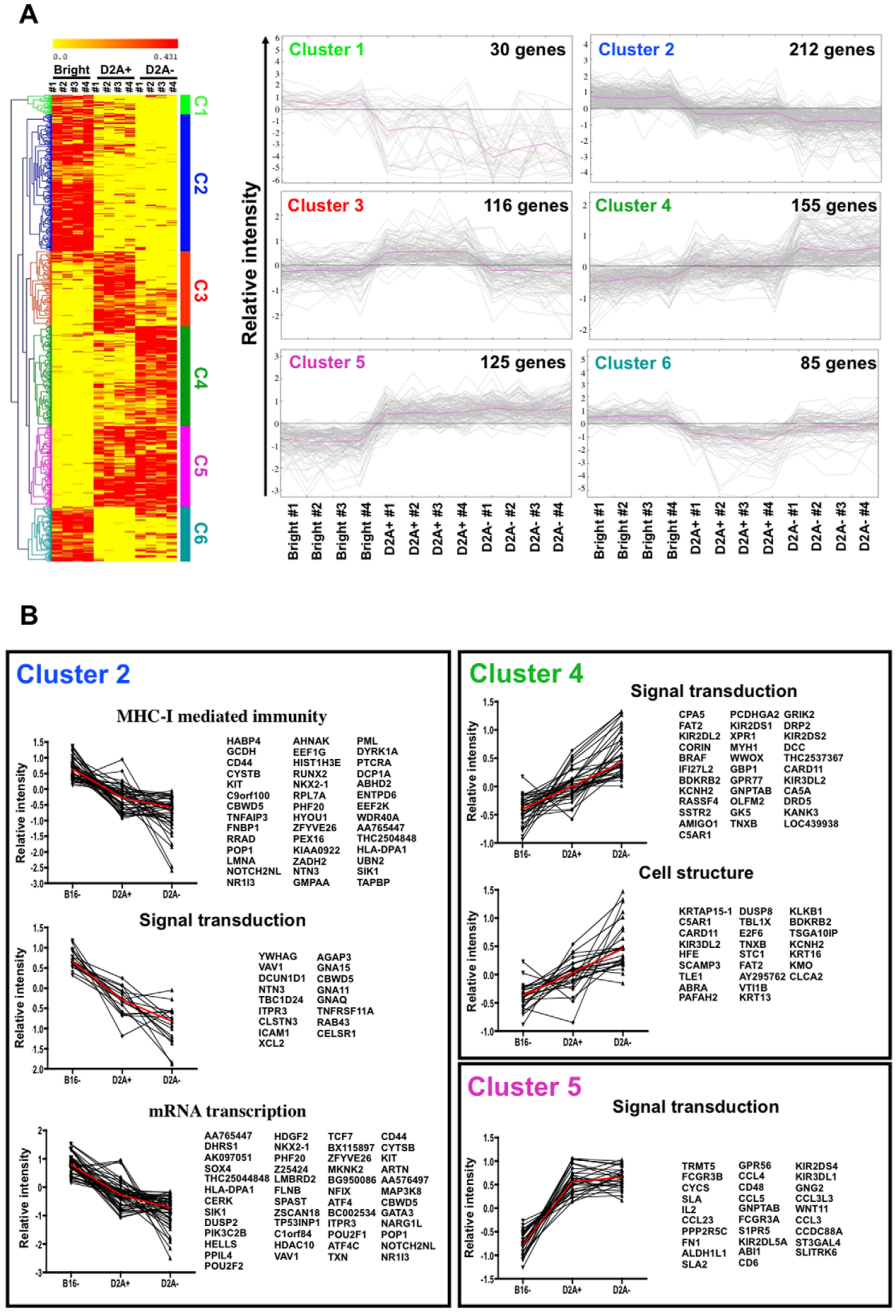
Whole genome microarray analysis. CD56**^bright^**CD16**^−^** (Bright), CD56**^dim^**NKG2A**^+^** (D2A+) and CD56**^dim^**NKG2A**^−^** (D2A−) NK cell subsets from 4 healthy donors (#1, #2, #3 and #4) were sorted and analysed using pan-genomic microarrays. (A) Left panel, Hierarchical clustering analysis of significantly modulated genes. The six clusters were numbered C1 to C6. Right Panel, alternative representation of each cluster showing the relative intensity of genes expression. Median relative intensities are represented by a red line. (B) Gene ontogeny analysis was performed with DAVID software on genes of clusters 2, 4 and 5. Highly significant terms are shown with the associated genes (boxes) and their expression profile in the three NK cell subsets. Median relative intensities are represented by a red line.

In an attempt to better characterize the different NK cell subsets, we compared the gene expression profiles of CD56^bright^, CD56^dim^NKG2A^+^, and CD56^dim^NKG2A^−^ NK cells. The analysis of variance revealed that 723 of the 41,000 genes tested showed significant changes in gene expression between the subsets at a p value (significance level) of p<0.01 (Table S1). The hierarchical clustering analysis of the differentially expressed genes revealed only six clusters (Fig. 2A). As expected, among the genes downregulated in cluster 1 (30 genes) and overexpressed in cluster 5 (125 genes) during the transition between CD56^bright^ to CD56^dim^, regardless of NKG2A expression, were some KIRs (Kir3DL1, Kir2DS4 and Kir2DL5A), other NK cell-surface receptors: (CD6, CD16a, 2B4, CD48 and CD62L), chemokines (CCL3, CCL4, CCL5, CCL3L3 and CCL23), and signalling molecules (STAT2, STAT4 and LAT2) (Table S2).

More interestingly, this gene profiling analysis largely confirmed that the CD56^dim^NKG2A^+^ subset mainly display an intermediary profile between CD56^bright^ and CD56^dim^NKG2A^−^ subsets. Indeed, the largest clusters, cluster 2 (212 genes) and cluster 4 (155 genes), corresponded to genes that were downregulated or overexpressed, respectively, according to the sequential CD56^bright^, CD56^dim^NKG2A^+^ and CD56^dim^NKG2A^−^ NK cell subsets. Focussing on these two major clusters, we used DAVID software (Table S2) to conduct a gene ontogeny analysis [20], [21]. As Fig. 2B shows, the most representative group of genes in cluster 2 involved mRNA transcription regulation. It contained notably GATA3, TCF7, VAV1 and SOX4, all described in T cells or NK activation or differentiation pathways [25], [26], [27]. Moreover, looking specifically at NK cell markers, we found substantial downregulation of both CD117, a key marker of immature NK cells, and of granzyme-K, a cytotoxic component of CD56^bright^ NK cells, thereby confirming our flow cytometry results (Fig. 1) (Table S2).

Conversely, cluster 4 was mainly characterised by the presence of genes involved in signal transduction and cell structure, including a number of tumour suppressor genes (FAT-2, DCC, RASSF-4 and BRAF) that play a role in the control of cell proliferation. In addition, expression increased for several genes in this cluster that are involved in the regulation of NK cell functional activities, such as KIRs (Kir2DL4, Kir3DL2, Kir2DS2, Kir2DL2 and Kir2DS1), granzyme-B and Ksp37 for cytotoxicity, and CARD11 (CARMA1) for the regulation of cytokine/chemokine production (Fig. 2B) (Table S2).

**Figure 3:**
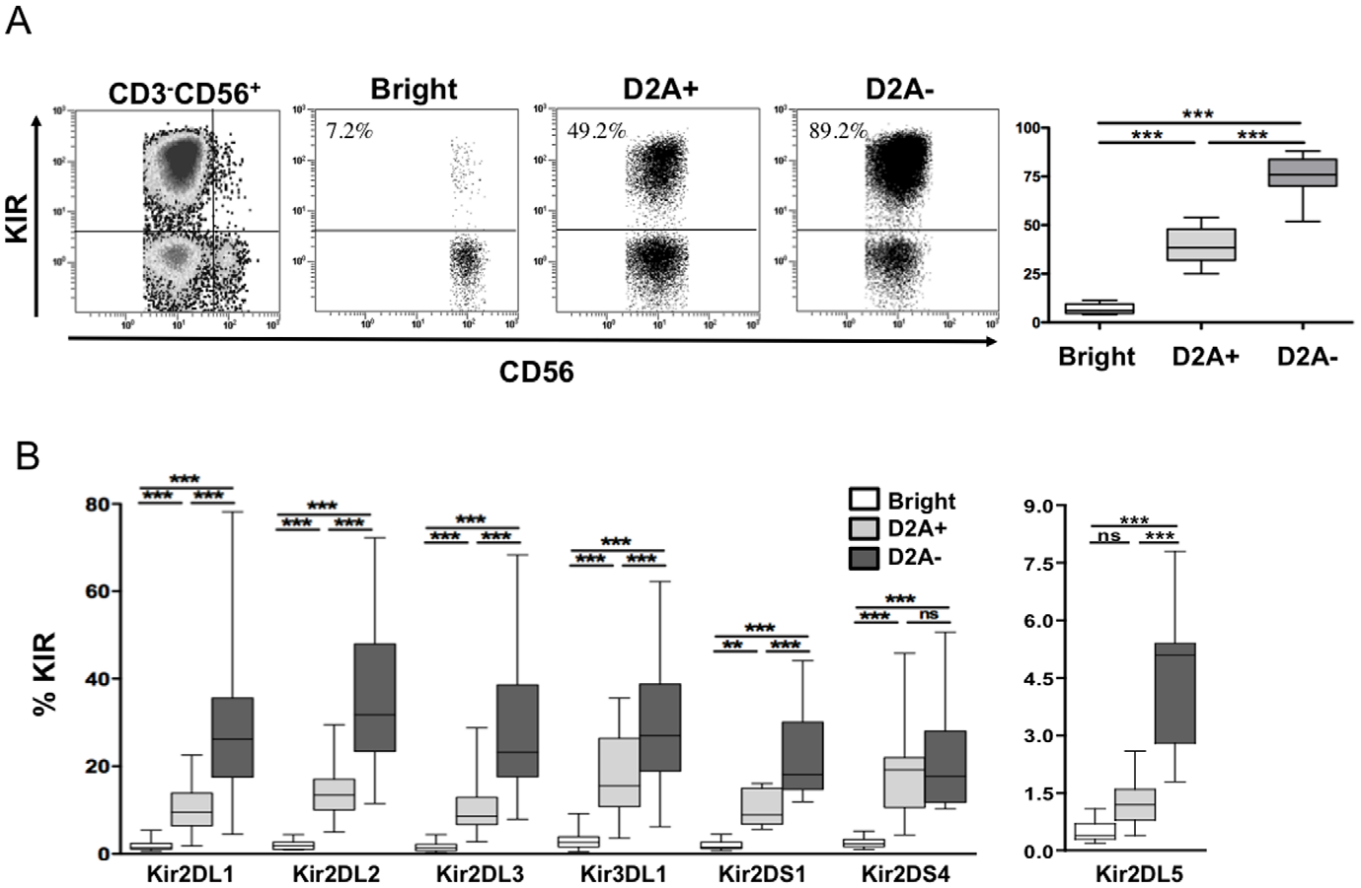
Extensive analysis of KIR expression. Cell surface expression of whole KIRs (A), or single KIR (B) on CD56^bright^ (open bars), CD56^dim^NKG2A^+^ (grey bars) and CD56^dim^NKG2A^−^ (black bars) NK cells subsets from 32 healthy controls. *: p<0,05; **: p<0.01; ***: p<0.001.

Although KIRs control the reactivity of mature NK cells, their expression also influences the functional maturation of developing NK cells. Fig. 3A shows that expression of whole KIRs, monitored by a cocktail of KIR antibodies, linearly increases among CD56^bright^, CD56^dim^NKG2A^+^ and CD56^dim^NKG2A^−^ subsets. More importantly, cell-surface expression of individual KIRs on all three NK cell subsets revealed that inhibitory (KIR2DL1, KIR2DL2, KIR2DL3, KIR3DL1, KIR2DL5) and activating (KIR2DS1) receptors increased significantly (p<0.001, for each marker) from the CD56^bright^ to CD56^dim^NKG2A^+^ subset and from the CD56^dim^NKG2A^+^ to CD56^dim^NKG2A^−^ subset (Fig. 3B). In contrast, KIR2DS4 rose only from the CD56^bright^ to CD56^dim^ independently of NKG2A expression. These data were consistent with the results obtained by gene array analysis (Fig. 2).

Together these results clearly show that specific combinations of NKG2A and CD56 expression permit to differentiate three phenotypicaly different subsets, including a CD56^dim^NKG2A^+^ subset displaying an intermediary profile between CD56^bright^ and CD56^dim^NKG2A^−^ subsets.

### NKG2A acquisition correlates with IFN-γ production in CD56^dim^NKG2A^−^ NK cells after IL12/IL18 stimulation

**Figure 4:**
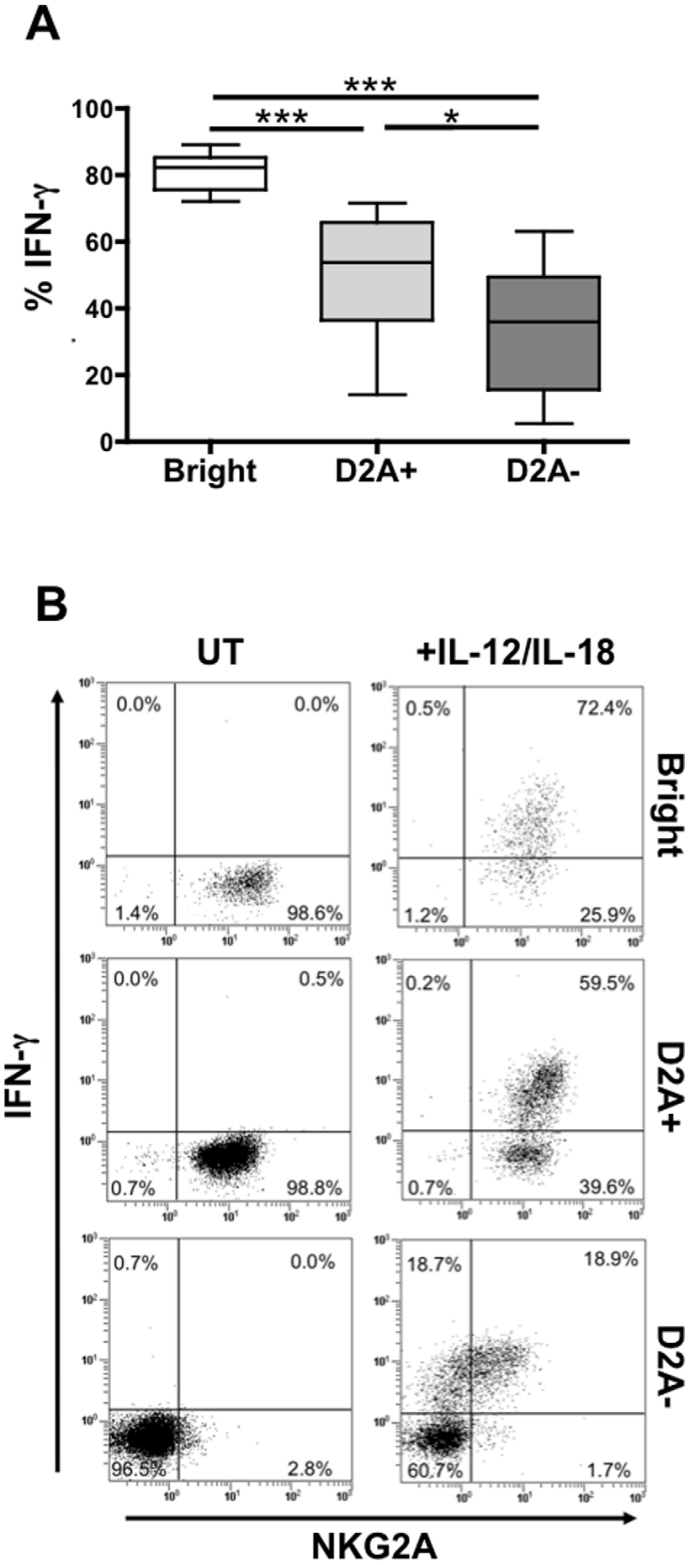
IFN-γ production is associated with NKG2A re-acquisition. (A) Intracellular expression of IFN-γ by CD56^bright^ (Bright), CD56^dim^NKG2A^+^ (D2A+) and CD56^dim^NKG2A^−^ (D2A−) NK cell subsets from 7 healthy donors, after cell sorting and IL-12/IL-18 overnight activation. *: p<0.05; **: p<0.01; ***: p<0.001. (B) Representative example of NKG2A expression and IFN-γ production, before (UT) and after stimulation with IL-12/IL-18 in CD56^bright^ (Bright), CD56^dim^NKG2A^+^ (D2A+) and CD56^dim^NKG2A^−^ (D2A−) NK cell subsets.

To determine the functional significance of these findings, we assessed intracellular IFN-γ production in highly purified CD56^bright^, CD56^dim^NKG2A^+^, and CD56^dim^NKG2A^−^ NK cell subsets after IL12+IL18 stimulation (Fig. 4). A significantly higher frequency of CD56^bright^ (compared with CD56^dim^) NK cells produced IFN-γ, regardless of whether or not the latter expressed NKG2A (p<0.001 in both cases). As Fig. 4A shows, around 80.9±6.2% of CD56^bright^ NK cells expressed IFN-γ, whereas only 49.7±20.8% of CD56^dim^NKG2A^+^ and 33.8±20.9% CD56^dim^NKG2A^−^ NK cells did so. Moreover, the number of IFN-γ positive cells decreased significantly in the CD56^dim^NKG2A^−^ NK cells, compared with the CD56^dim^NKG2A^+^ subset (p<0.05).

Next, we evaluated the effect of IL12+IL18 stimulation on the CD56^dim^NKG2A^−^ NK subset and found that a fraction of these cells re-expressed NKG2A (Fig. 4B). More intriguingly, up to 95% of this *de novo* subpopulation expressed IFN-γ, compared with 49.7±20.8% of the CD56^dim^NKG2A^−^ subset (Fig. 4B). Similar results were observed with CD94, but NKG2C, the activating counterpart of NKG2A, did not increase after IL12+IL18 stimulation (data not shown). In addition, CD56^bright^ and CD56^dim^NKG2A^+^ cells still expressed NKG2A after IL12+IL18 stimulation, regardless of their IFN-γ production (Fig. 4B). These results revealed that NKG2A could be specifically re-expressed on IFN-γ-producing-NKG2A^−^ NK cells, after IL-12/IL-18 stimulation.

### HLA-E expression on target cells restricts NK responsivity to the CD56^dim^NKG2A^−^KIR^+^ subset

**Figure 5:**
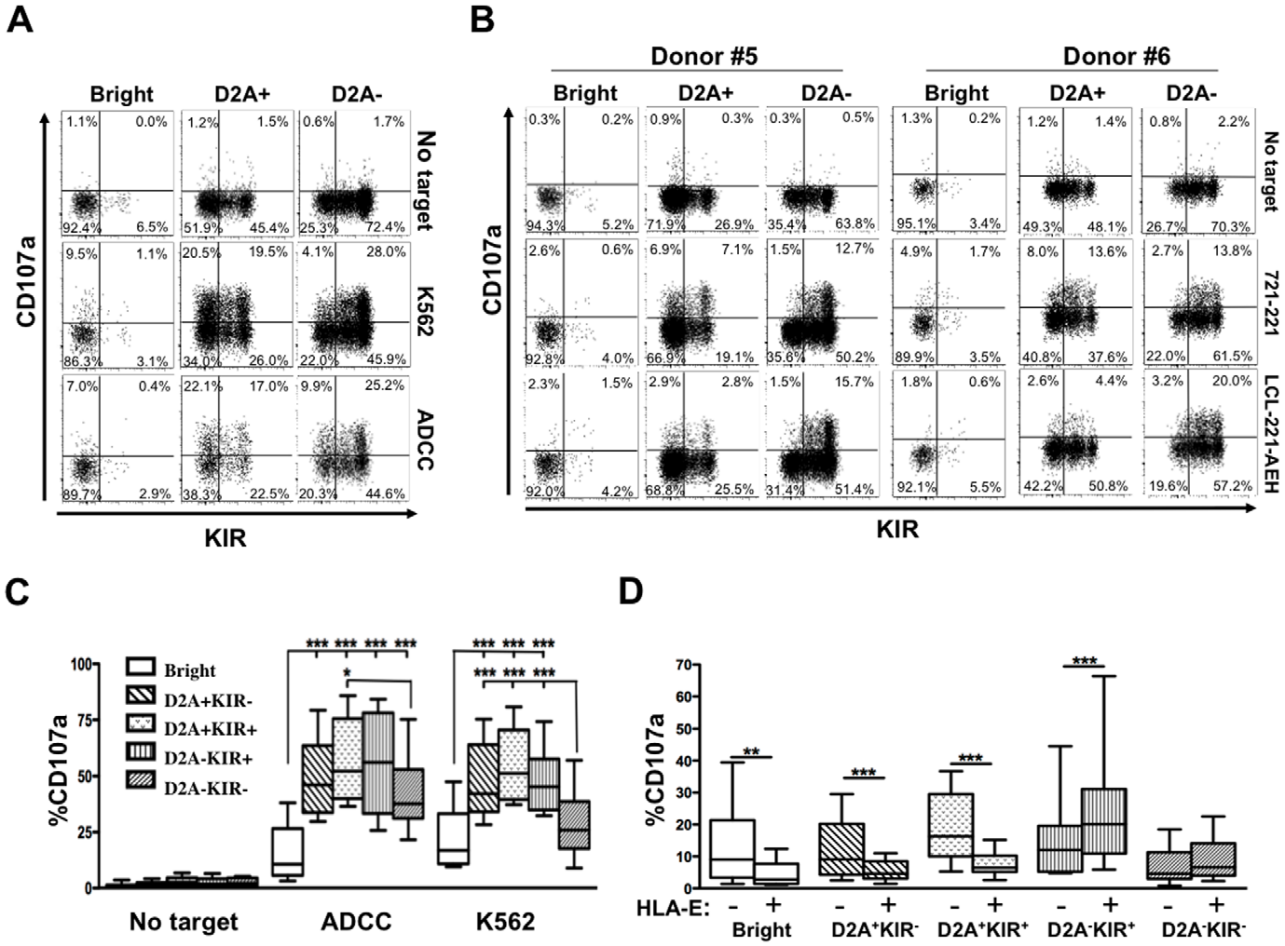
The CD56^dim^NKG2A^−^KIR^+^ NK cells are the only one able to degranulate against HLA−E+ target cells. CD107a degranulation assays of CD56^bright^ (Bright), CD56^dim^NKG2A^+^ (D2A+) and CD56^dim^NKG2A^−^ (D2A−) NK cell subsets were accessed without target, or against K562 or RAJI cells in the presence of 1 µg of rituximab (ADCC) (A), or against 721.221 target cells that do or do not express HLA-E (LCL-221-AEH) (C). Box and whiskers plots of CD107a degranulation capacities of CD56^bright^ (Bright), CD56^dim^NKG2A^+^KIR− (D2A+KIR−), CD56^dim^NKG2A^+^KIR^+^ (D2A+KIR+), CD56^dim^NKG2A^−^KIR^+^ (D2A−KIR+) and CD56^dim^NKG2A^−^KIR^−^ (D2A−KIR−) NK cell subsets from 11 healthy controls. NK cells were tested without target cells, against RAJI cells in the presence of 1 µg of rituximab (ADCC), or K562 cells (B) or 721.221 target cells that do or do not express HLA-E (D). *: p<0.05; **: p<0.01; ***: p<0.001.

Next we compared the degranulation capacity of these three subsets by assessing their ability to express CD107a at the cell surface. The response of CD56^bright^ NK cells to antibody-dependent cell mediated cytotoxicity (ADCC) was poor, but increased strongly in the CD56^dim^ subset, regardless of NKG2A expression (Fig. 5A). When we checked the degranulation of these NK cell subsets against HLA class-I negative K562 target cells, we observed that the direct cytotoxic capacity of CD56^bright^ NK cells was much lower than that of the CD56^dim^ subsets. More importantly, the level of CD107 expression was higher in CD56^dim^NKG2A^+^ than CD56^dim^NKG2A^−^ NK cells against K562 target cells (Fig. 5A).

Because KIRs play an established role in the education of NK cells, it was crucial to test their relative functional effects. Accordingly, we assessed the degranulation capacity of the following five NK cell subsets: CD56^bright^, CD56^dim^NKG2A^+^KIR^−^, CD56^dim^NKG2A^+^KIR^+^, CD56^dim^NKG2A^−^KIR^+^ and CD56^dim^NKG2A^−^KIR^−^. As Fig. 5A and 5C show, CD56^dim^NKG2A^−^KIR^−^ NK cells had significantly lower degranulation rates against K562 target cells than the other CD56^dim^ NK cell subsets (p<0.001), explaining that CD56^dim^NKG2A^−^ cells, regardless of KIR expression, are less able to degranulate against HLA class-I negative targets. Nonetheless, CD56^dim^NKG2A^−^KIR^−^ NK cells retained a significant capacity to respond to ADCC compared to the other CD56^dim^ subsets (Fig. 5A and 5C).

We next investigated whether degranulation of CD56^bright^, CD56^dim^NKG2A^+^KIR^−^, and CD56^dim^NKG2A^+^KIR^+^ NK cells was significantly inhibited in the presence of HLA-E-transfected 721.221 target cells, compared with the same cells against non-transfected 721.211 cells. Interestingly, CD56^dim^NKG2A^−^KIR^+^ cells were more functional against 721.221 target cells expressing HLA-E than against their non-transfected counterparts (Fig. 5B and 5D), which suggest preferential expression of NKG2C on CD56^dim^NKG2A^−^ cells. Taken together, functional data show that each CD56^dim^ NK cell expressing at least one inhibitory receptor is competent against HLA-I negative target cells. However, only the CD56^dim^NKG2A^−^KIR^+^ NK cells are competent to kill HLA-E-expressing target cells.

### Phenotypic changing correlate with stepwise decrease of NKG2A and acquisition of KIR, suggesting a new complex model of NK cells terminal differentiation

**Figure 6:**
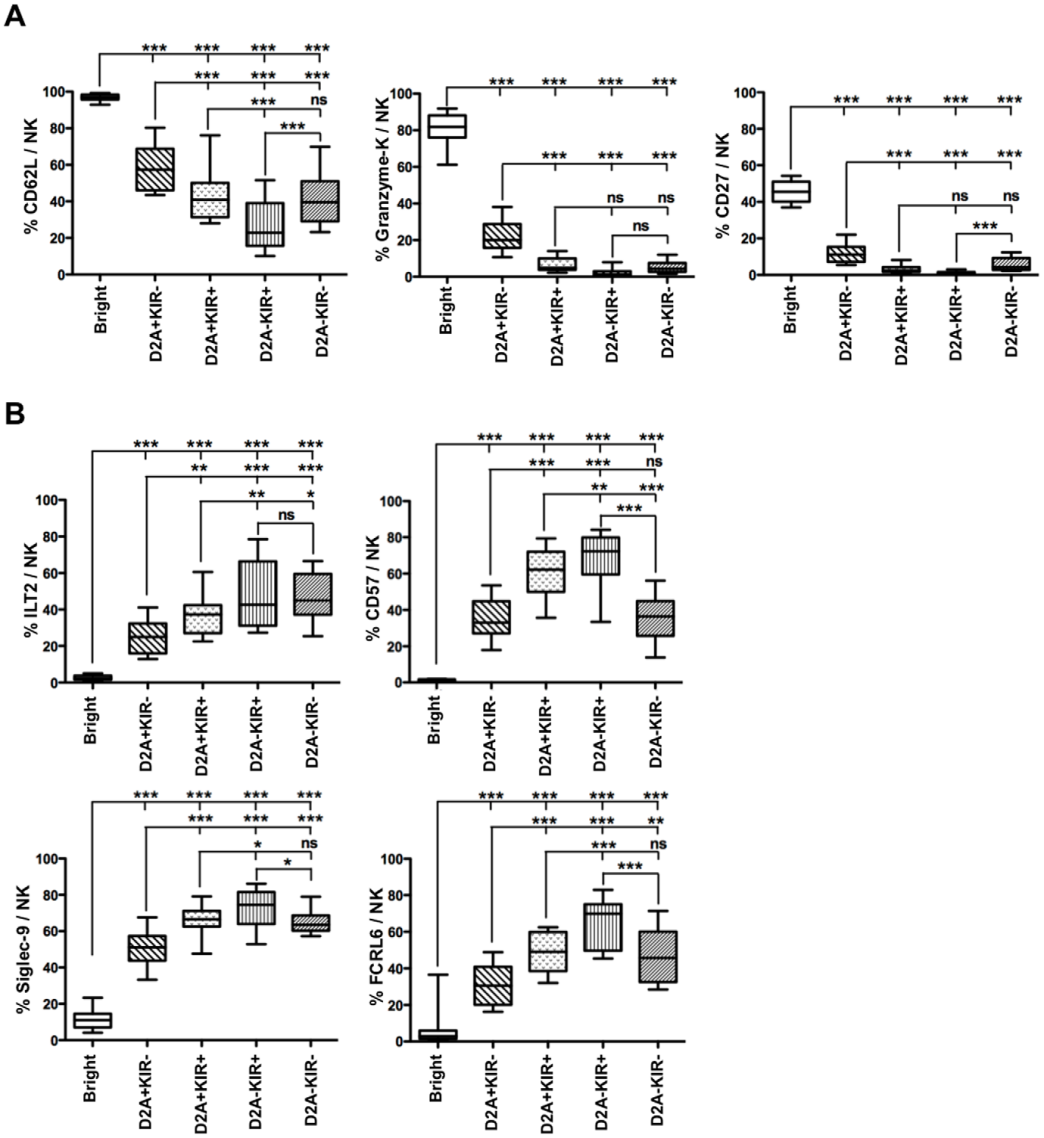
Stepwise decrease of NKG2A and acquisition of KIRs is shown by specific markers of NK cell maturation. Expression of CD62L, granzyme-K and CD27 (A) or ILT2, CD57, Siglec-9 and FCRL6 (B) on CD56^bright^ (Bright), CD56^dim^NKG2A^+^KIR^−^ (D2A+KIR−), CD56^dim^NKG2A^+^KIR^+^ (D2A+KIR+), CD56^dim^NKG2A^−^KIR^+^ (D2A−KIR+) and CD56^dim^NKG2A^−^KIR^−^ (D2A−KIR−) NK cell subsets from at least 16 healthy donors. *: p<0.05; **: p<0.01; ***: p<0.001.

**Figure 7:**
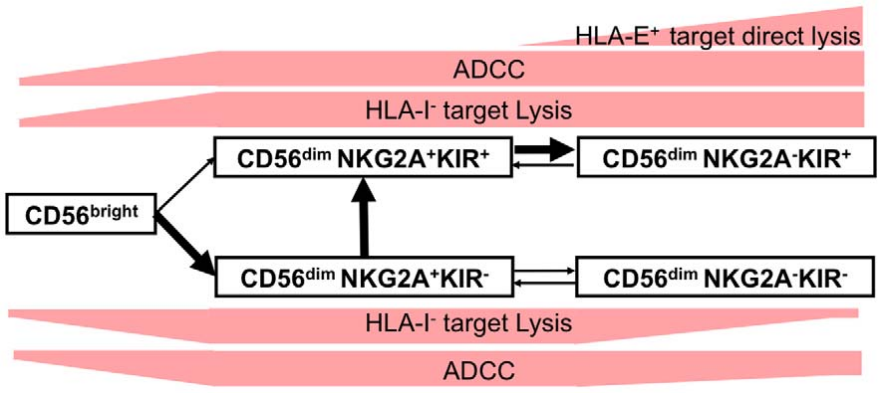
Model of terminal differentiation of NK cells. NK cell repertoire gradually changed from CD56^bright^ towards a CD56^dim^ dominant response; CD56^dim^NKG2A^+^KIR^−^ cells then develop into CD56^dim^NKG2A^+^KIR^+^ and then terminally differentiate into CD56^dim^NKG2A^−^KIR^+^ cells. Expression of KIR in a few CD56^bright^ NK cells also suggested that a part of those cells give rise directly to CD56^dim^NKG2A^+^KIR^+^. This model also includes the potential re-expression of NKG2A on CD56^dim^NKG2A^−^ NK cells after stimulation. Hyporesponsive CD56^dim^NKG2A^−^KIR^−^ NK cells are integrated as cells that failed to acquire KIR before NKG2A loss due to their incompletely differentiate phenotype.

Showing that KIRs coupled to NKG2A highlight functional subset specialization, we explored precisely the phenotype of these subsets. We looked markers that might provide clues about NK cell differentiation in the following five NK subsets: CD56^bright^, CD56^dim^NKG2A^+^KIR^−^, CD56^dim^NKG2A^+^KIR^+^ and CD56^dim^NKG2A^−^KIR^+^. Interestingly, the comparison of these subsets showed a continuum in the expression of some NK receptors, with a significant decrease of CD62L, CD27 and granzyme-K levels (Fig. 6A), with the progressive increase in markers associated with maturation, including ILT-2, CD57, Siglec-9 and FCRL6 (Fig. 6B). Finally, we analyzed in more detail the specific features of the hyporesponsive CD56^dim^NKG2A^−^KIR^−^ NK cell subset. Comparison between competent (NKG2A^−^KIR^+^) and hyporesponsive (NKG2A^−^KIR^−^) CD56^dim^ NK cells revealed that hyporesponsive cells expressed significantly more CD62L, and CD27 than competent cells. In contrast these hyporesponsive cells expressed less CD57, Siglec-9 and FCRL6 than competent NK cells (Fig. 6B). Together, these data support a new complex model of NK cell differentiation in which NKG2A expression, correlate inversely with KIRs expression (Fig. 7).

## Discussion

In this study, we set out to investigate the terminal differentiation of NK cells, focussing on NKG2A and KIRs as key markers during this dynamic process. Our data strongly endorse a new model of NK cell differentiation that begins with CD56^bright^ cells, all expressing high-density NKG2A^+^ and few KIRs, and evolves to a set of CD56^dim^NKG2A^−^KIR^+^ cells, passing through intermediate stages, each characterised by unique phenotypic and functional features. A part of our results agree with those in a recent report presenting a simple linear differentiation model based on the expression of CD94 [7]. However, differences in the expression pattern of other NK receptors have been noticed when comparing the inhibitory NKG2A^+^ and the activating NKG2C^+^ NK-cell subsets, which cannot be discriminated in CD94^+^ cells [28]. Furthermore, CD94 does not permit to conclude about specific activities, because NKG2A and NKG2C have opposite functions. The association reported between elevated numbers of circulating NKG2C^+^ NK cells and positive serology findings for viruses suggests that these NKG2A and NKG2C receptors might be involved in different stages of NK cell ontogenetic development [29], [30], [31]. Therefore this dichotomy, impossible when using CD94, is necessary for NK cells differentiation studies, particularly following pathogens encountering.

It is well established that CD56^bright^ NK cells produce more cytokines than CD56^dim^ NK cells in response to exogenous cytokine stimulation [32], [33]. We found that treatment with IL12/IL18 increased the frequency of IFN-γ production significantly more in CD56^bright^ NK cells than in CD56^dim^NKG2A^−^ NK cells: CD56^dim^NKG2A^+^ cells responded more vigorously in term of IFN-γ production than did CD56^dim^NKG2A^−^, but still significantly far less than CD56^bright^ NK cells, consistent with the recent data of Yu *et al* [7] on CD56^dim^CD94^+^ NK cells. Also, these results were in line with previous reports showing that CD56^dim^NKG2A^−^KIR^+/−^ NK cells have the developmental potential to reacquire NKG2A after cytokines stimulation [34], [35]. More importantly, our data provide evidence that the partial recovery of NKG2A correlates with IFN-γ production in CD56^dim^NKG2A^−^ cells after IL-12/IL-18 stimulation. Interestingly, Gustafson et al demonstrated that IFN-γ induces, through STAT-1 pathway, a high increase of HLA-E expression in exposed cells [36]. Therefore, IFN-γ production might induce its own regulation through HLA-E induction resulting in the inhibition of NKG2A expressing cells. These findings also indicate that CD56^dim^NKG2A^−^ phenotype does not represent an irreversible state for NK cells; they also highlight the robust plasticity of NK cell response to various stimuli.

Previous evidences support the hypothesis that NK cell development proceeds sequentially from CD56^bright^ to CD56^dim^ NK cells [5], [6], [7]. This model is buttressed by our data suggesting a robust link between the acquisition of functional capacities and both the stepwise decrease in NKG2A and the correlated acquisition of KIRs. Notably, we compared the CD56^bright^, CD56^dim^NKG2A^+^KIR^−^, CD56^dim^NKG2A^+^KIR^+^, and CD56^dim^NKG2A^−^KIR^+^ subsets and observed a gradient in the expression of NK markers between the three subsets, with a progressive decrease in CD62L, CD27 and granzyme-K, all highly expressed on CD56^bright^ cells from peripheral blood, together with a progressive increase in receptors associated with maturity, such as ILT-2, FCRL6, Siglec-9 and CD57. This notion of a continuum or gradient along a specific pathway of differentiation is perfectly illustrated by CD57 expression, which has been recognised as a marker of *in vitro* replicative senescence of T lymphocytes [37] and which is known to be absent on cord blood NK cells and subsequently increase with elderly [12]. Similarly, Cooley *et al* have shown that CD56^dim^KIR^+^ NK cells, which express high levels of CD57, proliferate more slowly [34]. This supports our model of NK cells terminal differentiation proposed in Fig. 7, in which the NK cell repertoire gradually changes from a CD56^bright^ toward a CD56^dim^ dominant response, whereby the CD56^dim^NKG2A^+^KIR^−^ subset develops first into a CD56^dim^NKG2A^+^KIR^+^ phenotype and then terminally differentiates into CD56^dim^NKG2A^−^KIR^+^ cells. As show by our functional data, these three CD56^dim^ subsets display the same ability to degranulate against HLA-I^−^ targets. More importantly, the acquisition of the terminal differentiated CD56^dim^NKG2A^−^KIR^+^ subset is crucial to acquire the capacity to respond against HLA-E^+^ target cells. Indeed, the degranulation of this subset is even increased by HLA-E expression on target cells. This particularity might be used in order to fight viruses like HCMV, which increase HLA-E expression on infected cells to escape immune system [38].

We also report the presence of a small population of CD56^bright^KIR^+^ NK cells, which has previously been described and which overlaps these developmental stages [39]. It has been suggested that this KIR expression on CD56^bright^ subset reflects their activation status in response to cytokines such as IL-15, which is responsible for homeostatic proliferation [34]. We propose as an alternative that a fraction of the CD56^dim^NKG2A^+^KIR^+^ cells might be directly derived from CD56^bright^ cells. Our results also show that a substantial fraction of CD56^dim^ NK cells that lacked both NKG2A and KIRs were hyporesponsive, even against the HLA class I-deficient K562 target cells; however, the paucity of available KIR mAb limited our study and prevents us from concluding definitively that KIR^−^ NK cells do not express any KIR. Intriguingly, we observed that these CD56^dim^NKG2A^−^KIR^−^ hyporesponsive cells had partially preserved their capacity for ADCC. This is consistent with their normal levels of perforin and granzyme-B cytotoxic components, as initially described [40]. It is likely that other receptors expressed by NK cells contribute to their education and may explain this activity. Recently Carrega *et al* [41] showed that hyporesponsive NK cells are fully responsive against autologous melanoma cells upon activation. In our study, expression of CD62L, CD27, CD57, Siglec-9 and FCRL6, suggests that CD56^dim^NKG2A^−^KIR^−^ NK cells are not fully differentiated, but can be re-educated under specific stimulation to become fully functional, in line with our model (Fig. 7). This is strong evidence that at least part of NK cell education might occur in the periphery during immune response, as suggest by Juelke *et al* [35].

Not surprisingly, multiple levels of tolerance keep fully functional NK cells in check. It is now well established that interaction between inhibitory receptors and their self-MHC class-I ligands affects NK cell education. Different mechanisms to maintain self-tolerance may accompany the different expression and effector function profiles of the various NK cell subsets. The mechanism underlying education of NK cells via inhibitory KIRs and NKG2A receptors remains unknown, and the ontogenetic development of tolerance is poorly understood. As shown in Fig. 7, our model, which is based on phenotypic and functional characteristics of the different NK cell subsets, neither refutes nor supports any particular mechanism for the regulation of self-tolerance, including licensing, arming, disarming or tuning/rheostat [16], [17], [18].

Taken together, our data shed new light on NK cell physiology and suggest a continuum of NK cell differentiation. The dynamics of this process seems to be closely linked to KIR acquisition and simultaneous NKG2A loss, and gives rise to NK cells responsive against HLA-E^+^ target cells. Our findings provide detailed insight into the plasticity of the NK cell repertoire and allow our model to include the failure of KIR acquisition before NKG2A loss in hyporesponsive NK cells.

## Supporting Information

Figure S1 NKG2C is expressed mainly separately from NKG2A among CD56^dim^CD94^+^ NK cells. (A) Expression of NKG2C, NKG2A and CD94 on NK cells from two representative healthy donors (#7, #8). (B) Expression of NKG2C on CD56^bright^ (Bright), CD56^dim^NKG2A^+^ (D2A+) and CD56^dim^NKG2A^−^ (D2A−) NK cell subsets from 38 healthy donors. Statistical analysis used one-way ANOVA with Tukey post-test. *: p<0.05; **: p<0.01; ***: p<0.001.
(8.37 MB TIF)

Table S1 Genes with significant modulations over the whole genome micro-arrays.
(0.22 MB XLS)

Table S2 Clusters extracted by complete hierarchical clustering and their associated ontogeny analysis using DAVID software.
(0.36 MB XLS)
