# Supplementary figures and images for: NK Cell Terminal Differentiation: Correlated Stepwise Decrease of NKG2A and Acquisition of KIRs
Source: PLoS One. 2010 Aug 6;5(8):e11966. doi: 10.1371/journal.pone.0011966 (PMC2917352; doi:10.1371/journal.pone.0011966)

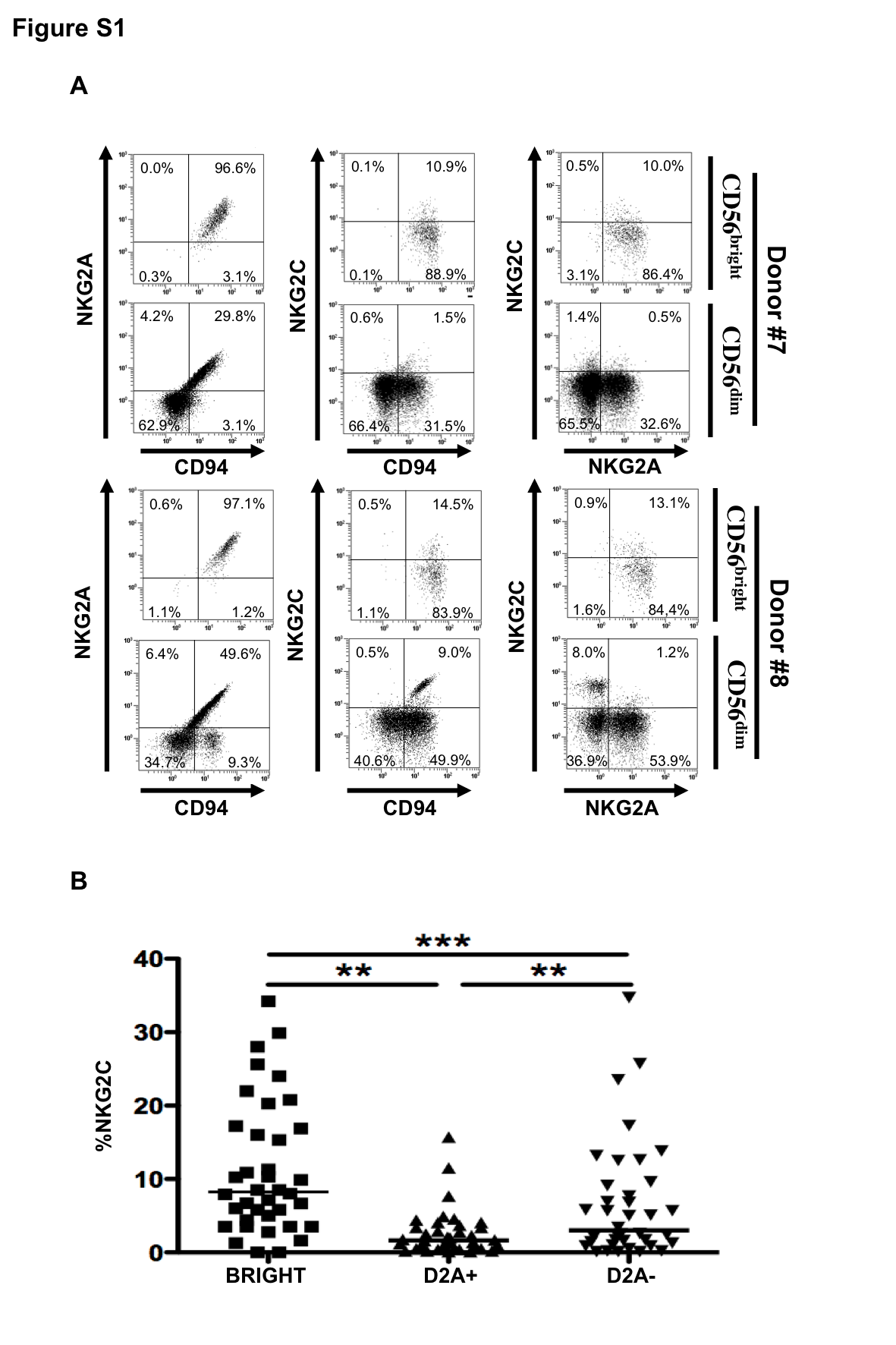

Supplement: Figure S1 — NKG2C is expressed mainly separately from NKG2A among CD56dimCD94+ NK cells. (A) Expression of NKG2C, NKG2A and CD94 on NK cells from two representative healthy donors (#7, #8). (B) Expression of NKG2C on CD56bright (Bright), CD56dimNKG2A+ (D2A+) and CD56dimNKG2A− (D2A−) NK cell subsets from 38 healthy donors. Statistical analysis used one-way ANOVA with Tukey post-test. *: p<0.05; **: p<0.01; ***: p<0.001. (8.37 MB TIF) [file pone.0011966.s001.tif]
